# Supplementary material for: Content-rich biological network constructed by mining PubMed abstracts
Source: BMC Bioinformatics. 2004 Oct 8;5:147. doi: 10.1186/1471-2105-5-147 (PMC528731; doi:10.1186/1471-2105-5-147)
Supplement: Additional File 5 — The original Chilibot query results of the term "long-term potentiation (LTP)" and 22 other terms, limiting the latest references analyzed to the years 1990, 1995, 2000, and 2004. [file 1471-2105-5-147-S5.bz2 › chilibotAdditionalFile5/ltp2000/html/SYNAPSIN I_PKA.html]

 


 **SYNAPSIN I** and **PKA** 
  
Found 6 abstracts in PubMed,  **6 abstracts were retrieved and analyzed**.  


---

 Search Google  |
 PDF files only 
|  EDU domain only 

---

**Interactive relationship** (e.g. stimulation, inhibition, etc)

**Parallel relationship** (e.g. studied together, co-existance, homology, etc.)

- **PKA** , CaM PK II and MLCK catalyzed the radiolabeling of histone 2A,  **synapsin I**  and myosin light chain MLC, known substrates for these kinases, respectively, yet no phosphate transfer to SCP2 was observed.  Ref: 2737166 Endocrinology, 1989
- The decrease in the concentration of CMK II which occurs in cytosol during synapse maturation was also observed in taxol polymerised microtubules and the effects of the change in the relative concentrations of CMK II and  **PKA**  on the phosphorylation of MAP 2 and  **synapsin I**  in this fraction were similar to that observed in the cytosol.  Ref: 1686473 Neurochem Res, 1991
- Transient expression of a CAT fusion gene under the control of the  **synapsin I**  promoter region is also inhibited by forskolin IBMX, as well as by protein kinase A  **PKA**  overexpression, suggesting that the decrease of  **synapsin I**  mRNA in response to forskolin IBMX is due to the inhibition of transcription.  Ref: 7711068 Biochim Biophys Acta, 1995
- Mutation of the CRE consensus does not affect the response to  **PKA** , but it reduces the constitutive activity of  **synapsin I**  promoter constructs down to 30 50%.  Ref: 7711068 Biochim Biophys Acta, 1995
- Similarly, the cAMP stimulated phosphorylation of the site on  **synapsin I**  labeled by the cAMP stimulated protein kinase  **PKA**  changed little during development whereas the calcium calmodulin stimulated phosphorylation of the CMK II site decreased dramatically in proportion to the decrease in the concentration of CMK II.  Ref: 1686473 Neurochem Res, 1991
